# Ecophysiological mechanisms underlying tomato spotted wilt of tobacco under climate change

**DOI:** 10.3389/fpls.2026.1935872

**Published:** 2026-09-25

**Authors:** Chunzhi Lu, Ming Liu, Junzi Zhu, Chengwei Yang, Yuanyuan Huang, Sheng Pu, Jiaen Su, Xingyu Zi, Yuling Zhang, Juan Li, Wenqiang Mei

**Affiliations:** 1 Yunnan Tobacco Company, Dali Prefecture Branch, Dali, Yunnan, China; 2 Hunan Agricultural University, Changsha, China

**Keywords:** climate change, ecophysiology, evidence synthesis, thrips vectors, tobacco, tomato spotted wilt

## Abstract

Tomato spotted wilt is an important viral constraint on tobacco production, but the direction and magnitude of future climate effects remain uncertain. This systematized narrative review evaluates evidence linking climate drivers with cultivated tobacco physiology, tomato spotted wilt virus (TSWV), thrips-vector ecology, weed reservoirs, surveillance, and management. Direct multifactorial experiments that simultaneously manipulate climate, TSWV, tobacco, and vector species are scarce; therefore, the synthesis explicitly separates direct tobacco evidence from evidence derived from other TSWV hosts, vector-only studies, general plant-stress physiology, and proposed mechanisms. Available tobacco studies show that long-term growth temperature, acute heat exposure, and progressive soil-water deficit alter photosynthesis, pigment metabolism, stomatal conductance, reactive oxygen species, and antioxidant responses, but they do not by themselves demonstrate enhanced TSWV replication or systemic movement. Field and vector studies indicate that weather can alter thrips development, survival, dispersal, and the temporal coincidence between viruliferous adults and susceptible tobacco, although responses are nonlinear and differ among *Frankliniella fusca*, *Frankliniella occidentalis*, *Thrips tabaci*, regions, and populations. We synthesize quantitative exposure ranges, identify confirmed and extrapolated pathways, compare diagnostic and surveillance options, and define a climate-adaptive management framework. The evidence supports region-specific risk assessment rather than a universal assumption that warmer or drier seasons will necessarily increase epidemics. Priority research requires factorial tobacco-TSWV-vector experiments, standardized physiological and virological endpoints, and independent validation of forecasting models.

## 1 Introduction

Climate change is altering the environmental context in which plant diseases develop through changes in mean temperature, short heat events, rainfall distribution, drought, atmospheric vapour-pressure deficit, and extreme weather (Canto et al., 2009; Pautasso et al., 2012; Singh et al., 2023; Angelotti et al., 2024; Lahlali et al., 2024; Verma et al., 2025). In vector-borne pathosystems, disease is not determined by the host, pathogen, and environment alone; vector survival, development, feeding, dispersal, and infection prevalence also determine whether inoculum reaches a susceptible crop (Canto et al., 2009; Bozinovic and Pörtner, 2015; Tsai et al., 2022; Jeger et al., 2023; Jangra et al., 2024). An ecophysiological framework is useful because it links plant function and stress responses with vector population processes and virus epidemiology, while also exposing where causal evidence is weak.

Cultivated tobacco (*Nicotiana tabacum* L.) is vulnerable to tomato spotted wilt, a disease caused by tomato spotted wilt virus (TSWV). TSWV has a broad host range and is transmitted in a persistent-propagative manner by several thrips species (Adkins, 2000; Parrella et al., 2003; Whitfield et al., 2005; Riley et al., 2011; Rotenberg et al., 2015; Rybicki, 2015; Güneş et al., 2022; Tiberini et al., 2025). In tobacco, early infection can cause chlorosis, ringspots, necrosis, deformation, stunting, and substantial loss of marketable leaf yield and quality (McPherson et al., 1999; Mila, 2011; Güneş et al., 2022). However, many mechanistic statements in the wider TSWV literature originate from tomato, pepper, ornamental crops, or general plant-stress experiments rather than from tobacco.

This review addresses four questions: (i) which climate-related responses are directly demonstrated in tobacco or tobacco-TSWV systems; (ii) how do major vector species and weed reservoirs differ among production regions; (iii) which proposed host-virus-vector pathways are supported only by extrapolation; and (iv) how can surveillance and management be adapted without transferring thresholds uncritically between regions. The scope is global, with emphasis on open-field tobacco and on the vector complexes most frequently reported in North America, Europe, and Asia. The framework is intended to complement, rather than replace, established reviews of orthotospovirus biology and thrips transmission (Whitfield et al., 2005; Riley et al., 2011; Rotenberg et al., 2015; Oliver and Whitfield, 2016; Tiberini et al., 2025).

The evidence-weighted conceptual framework used in this review is shown in **Figure 1**.

**Figure 1:**
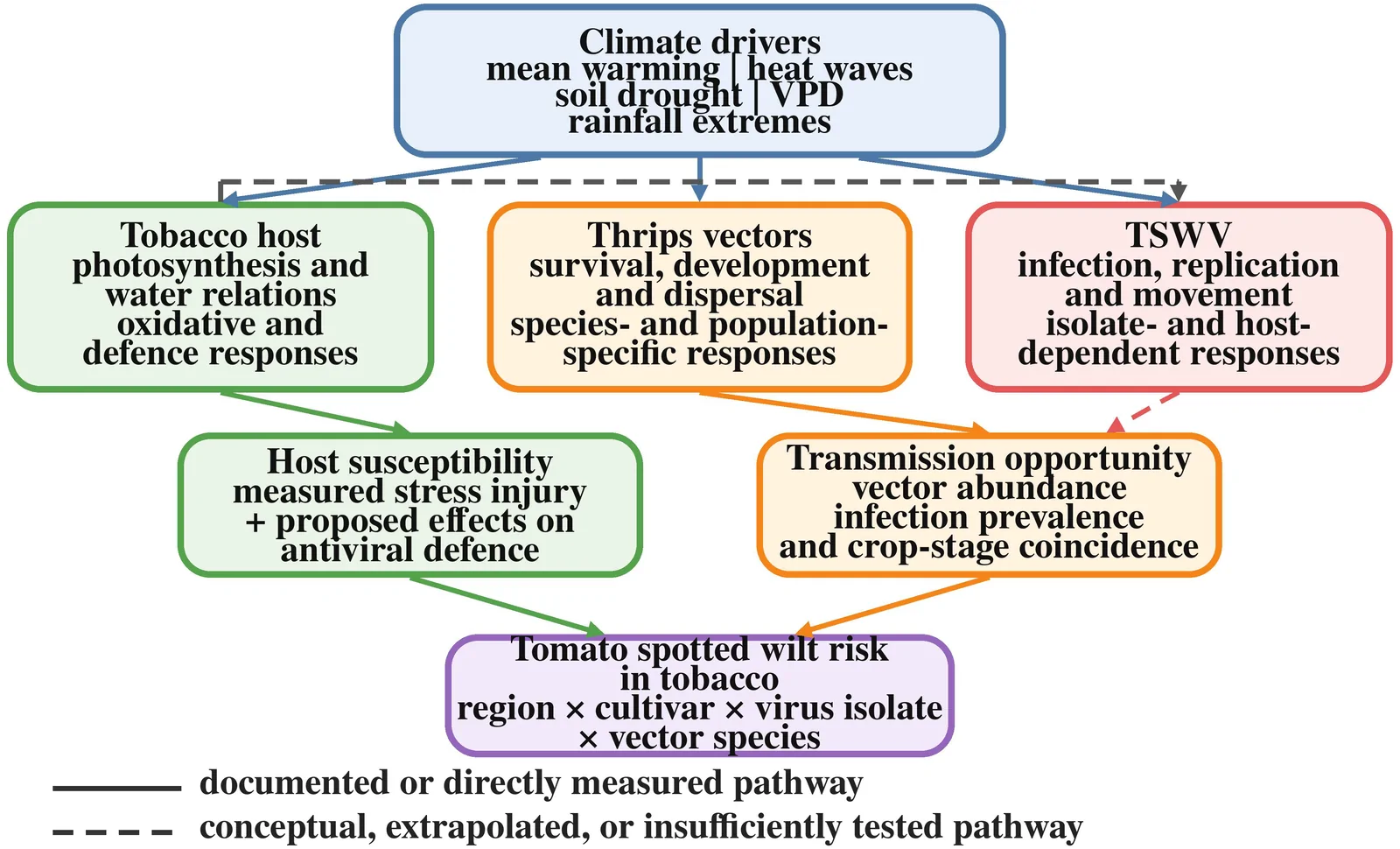
Evidence-weighted conceptual framework for tomato spotted wilt of tobacco under climate change. Solid arrows indicate documented or directly measured pathways; dashed arrows indicate conceptual, extrapolated, or insufficiently tested links. VPD, vapour-pressure deficit; TSWV, tomato spotted wilt virus.

## 2 Review methodology

A systematized narrative search was conducted and updated through 31 July 2026. PubMed, Web of Science Core Collection, Scopus, CAB Abstracts, and Google Scholar were searched using combinations of the terms “tobacco” OR “*Nicotiana tabacum*”, “tomato spotted wilt virus” OR “TSWV” OR “*Orthotospovirus tomatomaculae*”, “thrips” OR “*Frankliniella fusca*” OR “*Frankliniella occidentalis*” OR “*Thrips tabaci*”, and “temperature”, “heat”, “drought”, “humidity”, “rainfall”, “weed”, “diagnosis”, “forecast”, or “management”. Reference lists of key reviews and primary studies were also screened. Reporting followed the transparency principles of PRISMA 2020 where applicable to a systematized narrative review (Page et al., 2021).

English-language peer-reviewed studies and official taxonomic sources were included when they addressed tobacco, TSWV, a competent thrips vector, a confirmed or potential reservoir host, a climate exposure, surveillance, or management. Older foundational studies were retained when they established transmission biology, vector competence, or epidemiological mechanisms that have not been superseded. Studies were excluded when the pathogen, host, or vector was not relevant to the review question, when only unverified secondary claims were available, or when the cited result could not be linked to the conclusion. Screening consisted of duplicate removal, title and abstract assessment, full-text evaluation, and backward and forward citation tracking.

Evidence was classified as: direct tobacco-TSWV evidence; tobacco abiotic-stress evidence; direct vector or tobacco-field epidemiology; evidence from other TSWV hosts; or proposed mechanism. Because exposures, hosts, cultivars, vector populations, endpoints, and experimental settings were highly heterogeneous, effect estimates were not pooled. Conclusions were therefore weighted by biological relevance and directness rather than by vote counting. Representative evidence is summarized in **Table 1**.

**Table 1:** Representative evidence used to distinguish direct tobacco findings from extrapolated or proposed mechanisms.

Study and setting	Host/vector	Exposure or comparison	Main endpoints	Principal finding	Evidence class
controlled chambers (Yang et al., 2018)	*N. tabacum*	18.5, 23.5, and 28.5 °C mean growth temperature	Growth and plastid pigments	23.5 °C was most favorable under the tested non-stress conditions	Tobacco abiotic
controlled heat treatment (Tan et al., 2011)	*N. tabacum*	43 °C for 2 h	Gas exchange, PSII, antioxidants	Acute heat impaired photosynthesis; calcium pretreatment moderated injury	Tobacco abiotic
pot experiment (Su et al., 2017)	Flue-cured tobacco LJ851 and JX6007	Progressive water deficit for 2 weeks	Water status, photosynthesis, antioxidants, transcripts	Cultivars differed strongly in drought sensitivity	Tobacco abiotic
pot experiment (Zhang et al., 2024)	Q96, Z101, Y87, and Y116 tobacco	70-75%, 55-60%, 45-50%, and 35-40% soil moisture	Photosynthesis, fluorescence, ROS, MDA	Drought responses were severity- and cultivar-dependent	Tobacco abiotic
field survey (Güneş et al., 2022)	Tobacco, weeds, and field thrips	Natural field conditions in Türkiye	DAS-ELISA, incidence, weed hosts	31.2% of sampled tobacco and 10 weed samples were TSWV-positive	Direct tobacco field
weather-based model (Chappell et al., 2013)	Cultivated tobacco and *F. fusca* system	Weather, virus abundance, transmission intensity	Disease incidence	Weather acted through interacting inoculum and transmission processes	Direct tobacco epidemiology
laboratory experiment (Stumpf and Kennedy, 2005)	*F. fusca* on infected and non-infected hosts	18.3, 23.9, and 29.4 °C	Survival, size, development	Temperature, host, and isolate effects were interactive	Vector/TSWV
laboratory experiment (Katayama, 1997)	*F. occidentalis* on chrysanthemum	15, 20, 25, and 30 °C	Development and fecundity	Development accelerated with temperature; lower threshold was about 9.5 °C	Vector only
laboratory experiment (Jamieson et al., 2012)	*T. tabaci* on onion/leek substrates	12-30 °C	Development and feeding damage	Egg-to-adult development declined from 51.1 to 10.3 d; base threshold about 8.1 °C	Vector only
controlled studies (Maris et al., 2004; Shalileh et al., 2016; Nachappa et al., 2020)	Tomato or pepper with *F. occidentalis*	TSWV infection versus healthy host	Nutrition, defence, vector performance	TSWV altered host suitability and vector-associated traits in non-tobacco hosts	Other TSWV host

## 3 Pathogen characteristics and tobacco-specific impact

### 3.1 Terminology, taxonomy, and virus biology

Tomato spotted wilt is the disease name, whereas tomato spotted wilt virus is the English virus name and TSWV is its abbreviation. The corresponding ICTV species is *Orthotospovirus tomatomaculae*, placed in the genus *Orthotospovirus* and family Tospoviridae (Tiberini et al., 2025). Species and genus names are italicized; the virus name, abbreviation, and family name are not. The older generic term “tospovirus” is retained only in historical titles or when discussing literature published under the former taxonomy.

TSWV has a tripartite, negative- or ambisense single-stranded RNA genome comprising L, M, and S segments (German et al., 1992; Kormelink et al., 1992). The L segment encodes the RNA-dependent RNA polymerase; the M segment encodes glycoprotein and movement functions; and the S segment encodes the nucleocapsid and a nonstructural silencing-suppressor protein. Genome segmentation permits reassortment and contributes to variation among isolates in host adaptation, symptom severity, and resistance-breaking capacity (Kormelink et al., 1992; Pappu et al., 2009; Turina et al., 2016; Tiberini et al., 2025). TSWV is transmitted by competent thrips after acquisition during larval development and replication within the insect.

### 3.2 Field damage and limits of tobacco-specific evidence

Direct tobacco evidence consistently shows that early infection is more damaging than late infection. Infected leaves may develop chlorosis, necrotic lesions, ringspots, mosaic, puckering, and deformation, while severely affected plants show apical necrosis, shortened internodes, stunting, wilting, or death (McPherson et al., 1999; Mila, 2011; Güneş et al., 2022). Because tobacco leaf is the harvested product, reduced leaf expansion, uneven maturation, and necrosis directly lower marketable yield and cured-leaf quality.

Field surveys and epidemiological studies support associations among infection timing, vector activity, weed sources, weather, and final disease incidence (McPherson et al., 1999; Groves et al., 2002; Mila, 2011; Chappell et al., 2013; Mandadi and Scholthof, 2013; Güneş et al., 2022). By contrast, claims that TSWV directly reduces specific photosynthetic, hormonal, or metabolomic variables in cultivated tobacco are less well documented than analogous findings in tomato or pepper. Accordingly, this review treats detailed metabolic reprogramming and vector-mediated nutritional effects as plausible tobacco mechanisms when they are supported only by other TSWV hosts (Maris et al., 2004; Shalileh et al., 2016; Nachappa et al., 2020).

## 4 Transmission biology, vector species, and landscape sources

### 4.1 Acquisition, replication, and inoculation

TSWV transmission is stage-dependent and persistent-propagative. First- and second-instar larvae acquire virus while feeding on infected tissue; the virus infects the midgut, replicates, moves through the insect, and reaches the salivary glands (Ullman et al., 1992; Wijkamp et al., 1993; van de Wetering et al., 1996; Nagata et al., 1999; Moritz et al., 2004; Whitfield et al., 2005; Rotenberg et al., 2015; Chinnaiah et al., 2025). Adults that acquire virus for the first time usually do not become competent vectors because mature tissue barriers restrict movement to the salivary glands. Adults infected as larvae can inoculate healthy plants during probing and feeding, whereas stable transovarial transmission is generally absent (Jones, 2005; Whitfield et al., 2005).

Virus detection in a thrips does not automatically prove transmission competence. A positive molecular or serological result may indicate ingested or internalized virus, whereas competence requires successful larval acquisition, replication, salivary-gland infection, and subsequent inoculation (Nagata et al., 1999; Boonham et al., 2002; Jones, 2005; Whitfield et al., 2005; Rotenberg and Whitfield, 2018). This distinction is important when vector infection prevalence is used in warning systems.

The documented transmission sequence and the diagnostic caveat are summarized in **Figure 2**.

**Figure 2:**
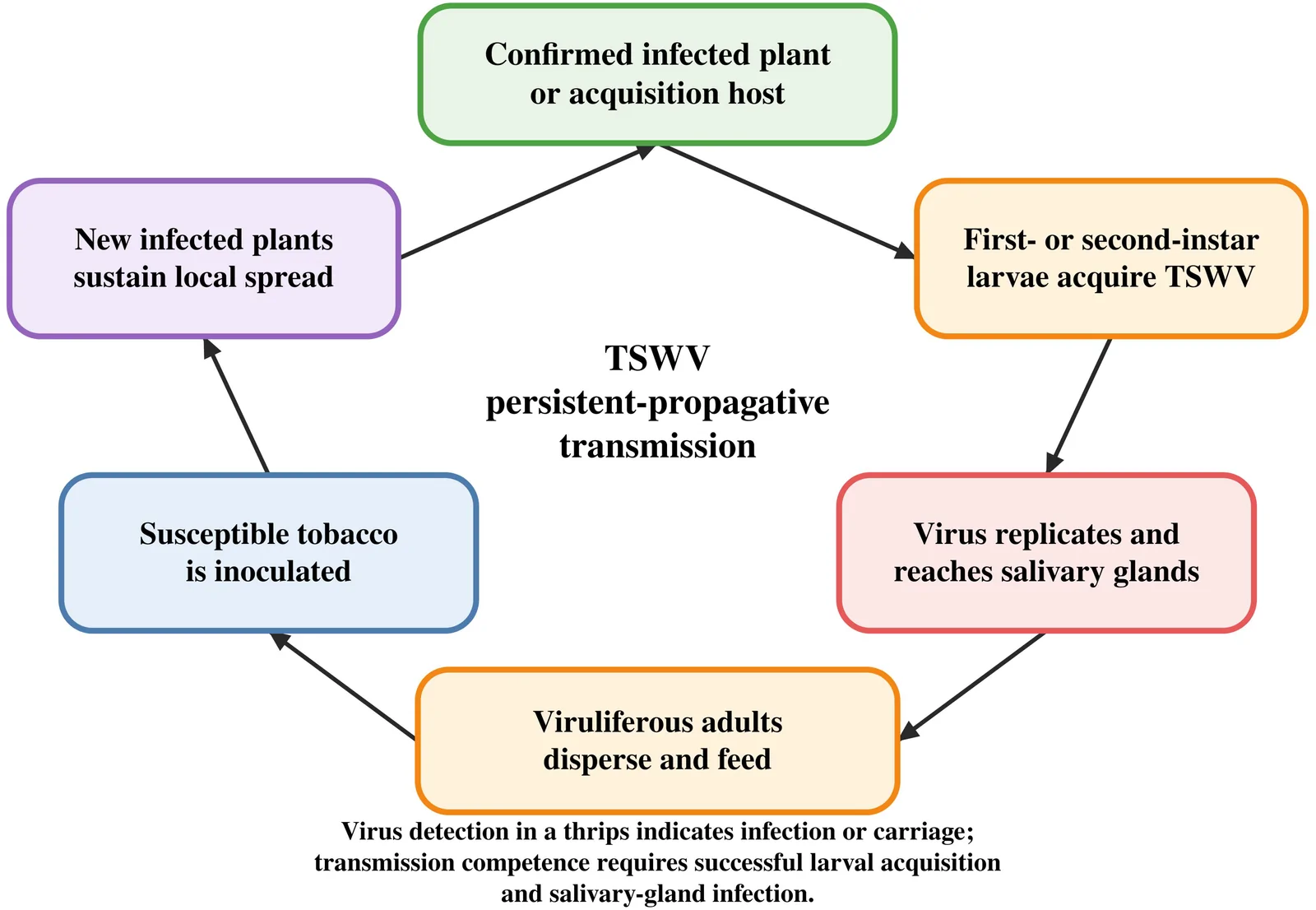
Field transmission cycle of TSWV mediated by thrips. Solid arrows denote established steps. Detection of viral RNA or antigen in an insect should not be interpreted as transmission competence unless acquisition stage and biological transmission are confirmed. TSWV, tomato spotted wilt virus.

### 4.2 Species-specific vector ecology

*Frankliniella fusca* is particularly important in open-field tobacco in the southeastern United States. Winter annual weeds and neighboring crops can support populations and TSWV sources, and spring temperature and precipitation influence population growth, dispersal, and virus movement (Groves et al., 2001, Groves et al., 2002, Groves et al., 2003; Stumpf and Kennedy, 2005; Morsello and Kennedy, 2009; Chappell et al., 2013; Goethe et al., 2022). Laboratory work at 18.3, 23.9, and 29.4 °C demonstrates that temperature effects on survival and development interact with host plant and virus isolate, so a single thermal optimum should not be generalized across populations (Stumpf and Kennedy, 2005).

*Frankliniella occidentalis* is globally distributed, highly polyphagous, and important in protected and open-field production systems. On chrysanthemum, pre-adult development required 34.2, 19.2, 12.1, and 9.5 d at 15, 20, 25, and 30 °C, respectively, with an estimated lower developmental threshold of 9.5 °C (Katayama, 1997). These values describe a specific host and population and should not be transferred directly to tobacco fields. Population or strain differences in fecundity and survival further limit universal predictions (Nielsen et al., 2021).

*Thrips tabaci* can transmit TSWV, but efficiency varies among populations with host preference and reproductive strategy (Chatzivassiliou et al., 2002). On onion or leek substrates, egg-to-adult development declined from 51.1 d at 12 °C to 10.3 d at 30 °C, and approximately 221 degree-days above 8.1 °C were required for development (Jamieson et al., 2012). These developmental data are useful for comparative context, not as tobacco-specific action thresholds.

### 4.3 Weed reservoirs and landscape context

Weed infection alone does not establish epidemiological importance. Surveys of weeds in Türkiye have also examined the host range of resistance-breaking TSWV variants (Fidan et al., 2024). A weed may be a virus host, a feeding host for adults, a reproductive host, a larval acquisition host, or a seasonal bridge host; these roles should be distinguished experimentally. Common chickweed (*Stellaria media*) can support *F. fusca* and TSWV movement in winter weed patches (Groves et al., 2001; Morsello and Kennedy, 2009). In tobacco fields in Türkiye, *Echinochloa crus-galli* and *Tordylium apulum* were detected as TSWV hosts, but detection alone does not demonstrate that they support larval acquisition or major field-to-crop transmission (Güneş et al., 2022).

Field margins, volunteer plants, preceding crops, ornamentals, and unmanaged vegetation can alter both source abundance and vector movement (Chatzivassiliou et al., 2001; Groves et al., 2001, Groves et al., 2002; Goethe et al., 2022; Güneş et al., 2022). Removal before transplanting can reduce source continuity, whereas sudden removal after vectors have accumulated may redirect adults into tobacco. Landscape conclusions must therefore be region-specific and based on both plant infection and vector reproduction or acquisition evidence.

## 5 Climate drivers and tobacco host ecophysiology

### 5.1 Mean warming and short heat events

Mean warming and short heat waves are biologically different exposures. In controlled tobacco studies, long-term mean temperatures of 18.5, 23.5, and 28.5 °C produced different growth and pigment responses, with 23.5 °C most favorable under the tested conditions (Yang et al., 2018). An acute treatment of 43 °C for 2 h impaired gas exchange and photosystem II performance (Tan et al., 2011). These experiments demonstrate temperature-dependent tobacco physiology, but neither study tested TSWV; they therefore cannot establish that warming increases viral replication or epidemic risk.

Heat can reduce photosynthetic carbon assimilation, destabilize chloroplast function, increase reactive oxygen species, and modify antioxidant and hormonal responses (Tan et al., 2011; Yang et al., 2018; Tan et al., 2020; Mittler et al., 2022). Under prolonged or severe stress, repair and detoxification may compete with growth and defence. Whether these changes facilitate TSWV infection, replication, or systemic movement in tobacco remains a testable hypothesis rather than a confirmed conclusion.

### 5.2 Soil drought, atmospheric dryness, humidity, and rainfall

Soil drought and high atmospheric vapour-pressure deficit should not be treated as interchangeable. Soil drought limits root water supply, whereas high vapour-pressure deficit increases atmospheric demand and may induce stomatal closure even when soil water is available. Drought can restrict photosynthesis through stomatal and mesophyll diffusion limitations, with metabolic injury becoming more important under severe stress (Flexas et al., 2004; Farooq et al., 2009). In a two-week progressive drought experiment, tobacco cultivars LJ851 and JX6007 differed in leaf water status, photosynthesis, antioxidant activity, and gene expression (Su et al., 2017). In a 2024 study, sufficient, mild, moderate, and severe water treatments were defined as 70-75%, 55-60%, 45-50%, and 35-40% soil moisture, respectively; photosynthesis, fluorescence, reactive oxygen species, and membrane injury changed with both severity and cultivar (Zhang et al., 2024).

These tobacco studies support cultivar-dependent physiological injury under water deficit, but they did not include TSWV or thrips. Statements that drought accelerates viral replication or systemic movement have therefore been reframed as hypotheses. Evidence from tomato and pepper indicates that virus-induced nutritional and defence changes can alter *F. occidentalis* performance (Maris et al., 2004; Shalileh et al., 2016; Nachappa et al., 2020), but corresponding tobacco experiments are still needed.

Rainfall effects can also be bidirectional. Heavy rain can reduce flight or dislodge small insects, while moderate rainfall can promote weed and crop growth and increase the availability of vector hosts (Morsello et al., 2008; Morsello and Kennedy, 2009). Drought-driven senescence may encourage movement from deteriorating plants, but the destination depends on landscape composition, crop phenology, irrigation, and vector species. **Table 2** summarizes quantitative exposures and the appropriate level of inference.

**Table 2:** Quantitative climate and vector responses relevant to the tobacco-TSWV pathosystem.

Driver or exposure	Reported range	Measured response	System studied	Interpretation for tobacco-TSWV risk
Long-term growth temperature	18.5, 23.5, 28.5 °C	Growth and pigment metabolism differed; 23.5 °C was most favorable	Tobacco only (Yang et al., 2018)	Direct host evidence; no virus inference
Acute heat	43 °C for 2 h	Reduced gas exchange and PSII performance	Tobacco only (Tan et al., 2011)	Direct heat injury; combined TSWV effect untested
Progressive soil drought	2 weeks	Cultivar-specific water, photosynthetic, antioxidant, and transcript responses	Tobacco only (Su et al., 2017)	Direct host evidence; no vector or virus
Soil moisture gradient	70-75% to 35-40%	Declining photosynthesis and fluorescence; increasing ROS and MDA	Four tobacco cultivars (Zhang et al., 2024)	Severity and cultivar dependence demonstrated
*F. occidentalis* temperature	15-30 °C	Development shortened from 34.2 to 9.5 d; base about 9.5 °C	Chrysanthemum (Katayama, 1997)	Vector-only benchmark; host/population dependent
*T. tabaci* temperature	12-30 °C	Development shortened from 51.1 to 10.3 d; base about 8.1 °C	Onion/leek (Jamieson et al., 2012)	Vector-only benchmark; not a tobacco threshold
*F. fusca* temperature and host	18.3, 23.9, 29.4 °C	Interactive effects on survival and development	TSWV, host plants, vector (Stumpf and Kennedy, 2005)	Supports nonlinear, context-dependent responses
Rainfall and spring weather	Field variation	Changed dispersal and population growth	Tobacco-region field studies (Morsello et al., 2008; Morsello and Kennedy, 2009)	Association is regional and may be bidirectional

## 6 Evidence for virus-host-vector interactions

### 6.1 Measured virus effects and extrapolation boundaries

Plant viruses can reprogram photosynthesis, metabolism, defence signaling, and resource allocation (Mandadi and Scholthof, 2013; Nicaise, 2014). In tomato and pepper systems, TSWV infection has altered host suitability, nutrients, defence pathways, and the performance of *F. occidentalis* (Maris et al., 2004; Shalileh et al., 2016; Nachappa et al., 2020). These studies provide mechanistic hypotheses for tobacco, but they are not direct evidence that infected tobacco undergoes the same metabolic changes or that those changes increase transmission.

Direct tobacco evidence is strongest for symptoms, incidence, yield-related damage, infection timing, vector occurrence, and weather-associated epidemiology (McPherson et al., 1999; Mila, 2011; Chappell et al., 2013; Mandadi and Scholthof, 2013; Güneş et al., 2022). Accordingly, the revised synthesis separates measured tobacco responses from extrapolated metabolic mechanisms. Claims about TSWV-induced changes in sugars, amino acids, phenolics, hormones, or vector preference are identified as evidence from other hosts unless tobacco was explicitly tested.

### 6.2 Confirmed and proposed combined-stress pathways

Confirmed components of the framework include: tobacco physiological responses to heat and drought; larval acquisition and persistent-propagative transmission; weather sensitivity of thrips development and dispersal; and the importance of vector infection prevalence, crop stage, and source hosts (Ullman et al., 1992; Wijkamp et al., 1993; van de Wetering et al., 1996; Nagata et al., 1999; Groves et al., 2001; Rizhsky et al., 2002; Groves et al., 2003; Moritz et al., 2004; Jones, 2005; Stumpf and Kennedy, 2005; Whitfield et al., 2005; Wahid et al., 2007; Morsello et al., 2008; Morsello and Kennedy, 2009; Reitz, 2009; Tan et al., 2011; Chappell et al., 2013; Rotenberg et al., 2015; Su et al., 2017; Rotenberg and Whitfield, 2018; Yang et al., 2018; Tan et al., 2020; Mittler et al., 2022; Chinnaiah et al., 2025). Proposed components include direct enhancement of TSWV replication by heat or drought in tobacco, predictable reallocation from antiviral defence under combined stress, and universal drought-driven movement into tobacco.

Interactions between abiotic stress and pathogen defence can alter disease susceptibility (Bostock et al., 2014). These proposed links are biologically plausible but may vary with cultivar, virus isolate, vector species, stress intensity, exposure sequence, and geographic population. They should therefore be framed as hypotheses for factorial experiments rather than as established causal conclusions. Future schematics and models should preserve this distinction between documented and conceptual pathways.

## 7 Surveillance, diagnostics, and risk forecasting

### 7.1 Diagnostic purpose, sample matrix, and limitations

Visual symptoms are insufficient for early or differential diagnosis. DAS-ELISA is practical for screening many plant samples but may be less sensitive when virus titre is low or tissue selection is poor. Conventional RT-PCR and RT-qPCR provide greater analytical sensitivity and sequence-specific confirmation, while real-time RT-LAMP offers rapid field-oriented detection when validated controls are included (Boonham et al., 2002; Mason et al., 2003; Caruso et al., 2024; Cayak and Fidan, 2024). Imaging and hyperspectral approaches can support non-destructive surveillance, but they require local calibration and laboratory confirmation because stress and other diseases can generate similar spectral responses (Mensah et al., 2024; Mao et al., 2025).

Plant and insect matrices answer different questions. A positive plant test confirms infection of the sampled tissue. A positive result from an individual or pooled thrips sample indicates viral RNA or antigen in the insects, but not necessarily biological transmission competence (Nagata et al., 1999; Boonham et al., 2002; Jones, 2005; Whitfield et al., 2005; Rotenberg and Whitfield, 2018). Individual-thrips RT-qPCR is useful for estimating infection prevalence, whereas pooled testing is more economical but reduces information about individual variation.

### 7.2 From surveillance data to actionable risk categories

Risk assessment should combine at least four dimensions: vector abundance, the proportion of TSWV-positive thrips, abundance and status of source hosts, and overlap between vector activity and susceptible tobacco stages (Groves et al., 2001; Mila, 2011; Chappell et al., 2013). Weather variables should modify these components rather than act as independent universal triggers. For example, the same warm period can increase development but also reduce survival if temperatures exceed the local optimum.

A practical warning system can classify risk as low, moderate, or high using locally validated thresholds and predefined actions. Low risk may justify routine surveillance; moderate risk may trigger intensified plant and vector testing and nursery protection; high risk may trigger coordinated source suppression, transplant-timing adjustments, exclusion, and targeted vector management. Thresholds require independent validation for each region, cultivar, and dominant vector complex.

The revised surveillance and decision pathway is shown in **Figure 3**.

**Figure 3:**
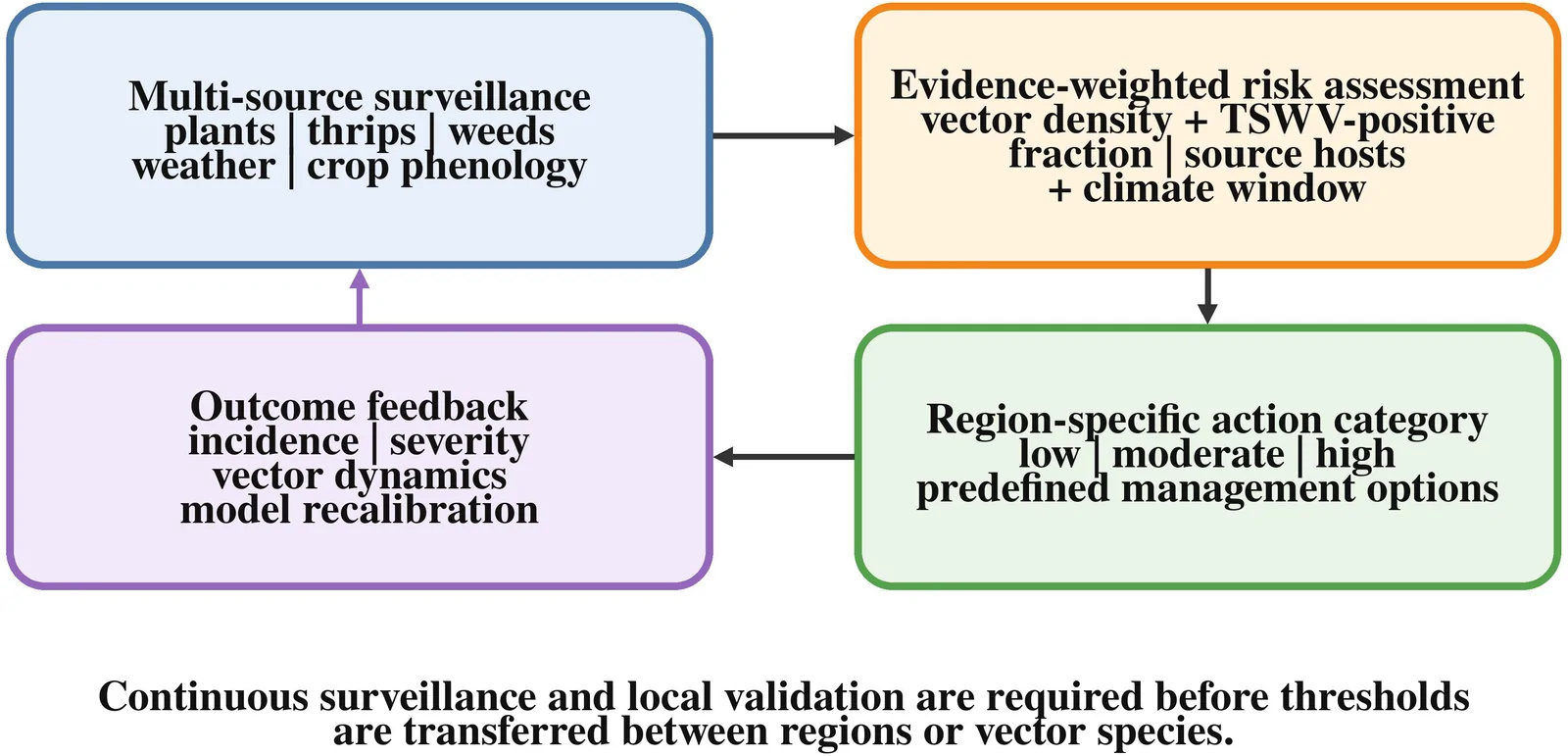
Climate-adaptive surveillance, evidence-weighted risk assessment, management decisions, and outcome feedback. Thresholds must be validated locally and should combine vector abundance, TSWV-positive fraction, source hosts, crop phenology, and weather. TSWV, tomato spotted wilt virus.

## 8 Climate-adaptive integrated management

Established measures include production of virus-free transplants, exclusion or suppression of thrips in nurseries, pre-transplant management of infected source plants, monitoring of vector flights, and integration of resistant or tolerant germplasm where available (Groves et al., 2002; Momol et al., 2004; Mila, 2011; Riley et al., 2011; Chappell et al., 2013; Mandadi and Scholthof, 2013; Turina et al., 2016; Qi et al., 2021). Transplant timing can reduce phenological overlap with major vector flights, but the optimal date is regional and should be based on local weather and vector records.

Sticky traps are monitoring tools rather than universal treatment thresholds. Trap color and capture efficiency vary with species, crop background, and environment. Irrigation can reduce drought injury, and balanced nitrogen may avoid excessively succulent growth, but neither practice should be described as a proven TSWV control unless tobacco-specific disease data are available. Biological control and physical barriers are promising, especially in nurseries or protected systems, but performance depends on climate and production setting.

Chemical control has important limitations. Incoming viruliferous adults can inoculate plants during brief probes before mortality, and eggs, concealed larvae, and soil-associated pupal stages are incompletely exposed. Insecticides may reduce local population growth or secondary spread, but repeated prophylactic applications do not guarantee prevention of primary infection and can select resistance or disrupt natural enemies (Whitfield et al., 2005; Riley et al., 2011; Rotenberg et al., 2015; Turina et al., 2016). Applications should therefore be guided by local surveillance, vector species, resistance-management principles, and label requirements. **Table 3** summarizes the surveillance, diagnostic, and management components and their principal limitations.

**Table 3:** Surveillance, diagnostic, and management components with their principal limitations.

Component or method	Matrix or indicator	Primary use	Important limitation	Decision role
DAS-ELISA	Symptomatic or systematically sampled plant tissue	High-throughput plant screening	Sensitivity depends on titre and tissue quality	Confirm field infection and reservoir status
RT-PCR/RT-qPCR	Plant tissue or individual/pool thrips	Sensitive, sequence-specific detection	Positive thrips result does not by itself prove competence	Estimate infection prevalence and confirm diagnosis
Real-time RT-LAMP	Plant tissue; validated field extracts	Rapid near-field detection	Requires contamination control and local validation	Rapid triage before confirmatory testing
Sticky traps	Adult thrips captures	Flight timing and relative abundance	Colour and counts are species- and setting-dependent	Identify local activity peaks
Weed survey	Species, infection, larval presence	Identify potential sources and bridge hosts	Infection alone does not prove acquisition-host importance	Prioritize source-management studies
Weather monitoring	Temperature, rainfall, humidity, soil moisture, VPD	Define exposure windows	Mean values can obscure extremes and nonlinear responses	Modify locally validated risk models
Insecticides	Target vector stage and local population	Reduce population growth or secondary spread	May not prevent rapid inoculation by immigrants	Use selectively within IPM
Irrigation and nutrition	Plant water and nutrient status	Maintain crop vigor	Not proven as direct TSWV suppression in most regions	Support stress-resilient crop management

## 9 Knowledge gaps and research priorities

Priority experiments should use factorial designs that manipulate at least temperature regime (control, chronic warming, short heat wave), water regime (well-watered, soil drought, high VPD where feasible), TSWV status, and vector species or population. Experiments should include multiple tobacco cultivars and virus isolates and should record the sequence and duration of exposures rather than only final stress categories.

Core host endpoints should include gas exchange, chlorophyll fluorescence, leaf water potential, stomatal conductance, membrane injury, reactive oxygen species, antioxidant enzymes, defence hormones, targeted metabolites, viral titre, and systemic movement. Vector endpoints should include acquisition rate, within-vector viral load, salivary-gland infection, inoculation efficiency, probing behavior, survival, development, fecundity, and dispersal. Detection of viral RNA should be separated from demonstrated transmission.

Field studies should connect plot-scale measurements with landscape composition, weed phenology, source-host competence, vector infection prevalence, and crop stage. Forecasting models should be trained and independently validated across years and regions, report uncertainty, and test whether adding physiological or vector-infection variables improves predictions beyond weather and abundance alone. Long-term surveillance networks are needed to determine whether climate-driven changes are directional, episodic, or offset by nonlinear heat and rainfall effects.

## 10 Conclusions

Tomato spotted wilt of tobacco is governed by interactions among TSWV, tobacco, thrips vectors, source hosts, crop phenology, and weather. Direct evidence supports climate sensitivity of tobacco physiology and thrips population processes, but tobacco-specific experiments that connect those responses to viral replication and transmission remain limited. Consequently, much of the proposed ecophysiological framework is inferential and should not be interpreted as a universal prediction that warmer or drier conditions will increase disease.

Risk is expected to depend on region, cultivar, virus isolate, vector species and population, landscape context, exposure intensity, and timing. Near-term progress requires transparent evidence classification, species-specific and quantitative synthesis, improved plant and vector diagnostics, and locally validated forecasting and management. These steps can move disease control from broad narrative expectations toward testable mechanisms and climate-adaptive decision support.
